# A Molecular Smart Surface for Spatio-Temporal Studies of Cell Mobility

**DOI:** 10.1371/journal.pone.0118126

**Published:** 2015-06-01

**Authors:** Eun-ju Lee, Wei Luo, Eugene W. L. Chan, Muhammad N. Yousaf

**Affiliations:** 1 Department of Chemistry and the Carolina Center for Genome Science, University of North Carolina at Chapel Hill, North Carolina, United States of America; 2 Department of Chemistry and Biology, Centre for Research in Biomolecular Interaction, York University, Toronto, Ontario, Canada; National Center for Scientific Research Demokritos, GREECE

## Abstract

Active migration in both healthy and malignant cells requires the integration of information derived from soluble signaling molecules with positional information gained from interactions with the extracellular matrix and with other cells. How a cell responds and moves involves complex signaling cascades that guide the directional functions of the cytoskeleton as well as the synthesis and release of proteases that facilitate movement through tissues. The biochemical events of the signaling cascades occur in a spatially and temporally coordinated manner then dynamically shape the cytoskeleton in specific subcellular regions. Therefore, cell migration and invasion involve a precise but constantly changing subcellular nano-architecture. A multidisciplinary effort that combines new surface chemistry and cell biological tools is required to understand the reorganization of cytoskeleton triggered by complex signaling during migration. Here we generate a class of model substrates that modulate the dynamic environment for a variety of cell adhesion and migration experiments. In particular, we use these dynamic substrates to probe in real-time how the interplay between the population of cells, the initial pattern geometry, ligand density, ligand affinity and integrin composition affects cell migration and growth. Whole genome microarray analysis indicates that several classes of genes ranging from signal transduction to cytoskeletal reorganization are differentially regulated depending on the nature of the surface conditions.

## Introduction

Cells do not live in static surroundings, they exist in highly evolving dynamic environments [1–2]. During cell adhesion and migration, cells adapt and communicate to their environment by numerous methods ranging from differentiation, gene expression, growth and apoptosis [3–9]. How and when cells determine to adhere and migrate is important to a number of fundamental biological processes such as wound healing, metastasis, inflammation and development [10–13]. In order to elucidate the spatial and temporal mechanisms of those complex processes on a molecular basis, model substrates that can be dynamically modulated where the interaction between cell and material is defined at the molecular level would be extremely useful [14–16]. Herein, we develop a novel surface chemistry technology to generate a class of molecularly well-defined dynamic substrates that permit the precise modulation of environment that an adherent cell senses in space and time. We demonstrate this methodology by electrically switching on adhesive ligands that induce the migration and growth of cells, which were initially confined on the defined patterns. We determine how the interplay of several parameters including the population of cells, pattern geometry, ligand density, ligand affinity and integrin composition influence cell behavior on these dynamic surfaces. We also found that cells retain an imprint of their initial condition, which influences the subsequent migratory behavior as if cells have a memory of earlier environment. Genome-wide microarray analysis revealed that several genes in signal transduction, cytoskeletal reorganization and proliferation are differentially regulated at the transcription level depending on the dynamic surface microenvironment.

The extracellular matrix (ECM) is a highly dynamic, insoluble aggregate of collagens, proteoglycans, structural glycoproteins, and elastin that provides structural support for the adhesion, growth, differentiation, migration, and survival of mammalian cells. For a cell to undergo migration, it must first adhere to another cell or the ECM through cell surface receptor-ligand interactions. Integrins and syndecans, which are transmembrane proteins, represent the most common cell surface receptor families that facilitate cell adhesion to the ECM and transduce extra- and intracellular signals. Fibronectin (FN) is a predominant ECM glycoprotein that contains three homologous globular domains: type I, II, and III, and possesses a number of interaction sites for both integrins and syndecans. As such, FN plays an important role in cell adhesion, growth, migration, and differentiation and is critical to cellular processes, including embryogenesis and tissue repair. A number of cell types bind to FN regions that span the 8th to 10th type III (FNIII_8–10_) cell-binding domain. Arg-Gly-Asp (RGD), found in FNIII_10_, was identified as the minimal cell attachment sequence of α5β1 and αVβ3 integrin recognition. To prepare surfaces for the dynamic study of complex cell behavior we designed model substrates based on the following considerations: 1. The surface must be able to present ligands in well-defined environment and must be amenable to chemoselective reactions that immobilize ligands or transformations that reveal ligands to adherent cells during the course of cell migration or cell adhesion. This important feature requires an orthogonal chemical reaction to immobilize ligands to a surface in the presence of cells and complex protein mixtures with no side reactions. The immobilization reaction should be fast, kinetically well-behaved, and unreactive toward other biopolymers (DNA, RNA, proteins, lipids etc.) at physiological conditions. 2. The yield of immobilization reaction and therefore density of immobilized ligands on the surface must be precisely determined. This requires that the model substrates are compatible with sensitive and quantitative in situ surface analytical techniques. 3. The surface should have the ability to pattern different population of cells in defined geometries ranging from a single cell to hundreds of cells. 4. A non-invasive method that can activate the surface by immobilizing or unveiling ligands in the presence of attached cells is required. 5. The surface must be inert to non-specific protein adsorption, i.e. the only interaction between cell and material is a ligand-receptor mediated interaction. 6. The model substrate should be compatible with high throughput microarray technologies and several surface and microscopy techniques that are routinely used to characterize cell behavior and 7. The surface should possess the ability to perform massively parallel experiments simultaneously.

**Fig 1:**
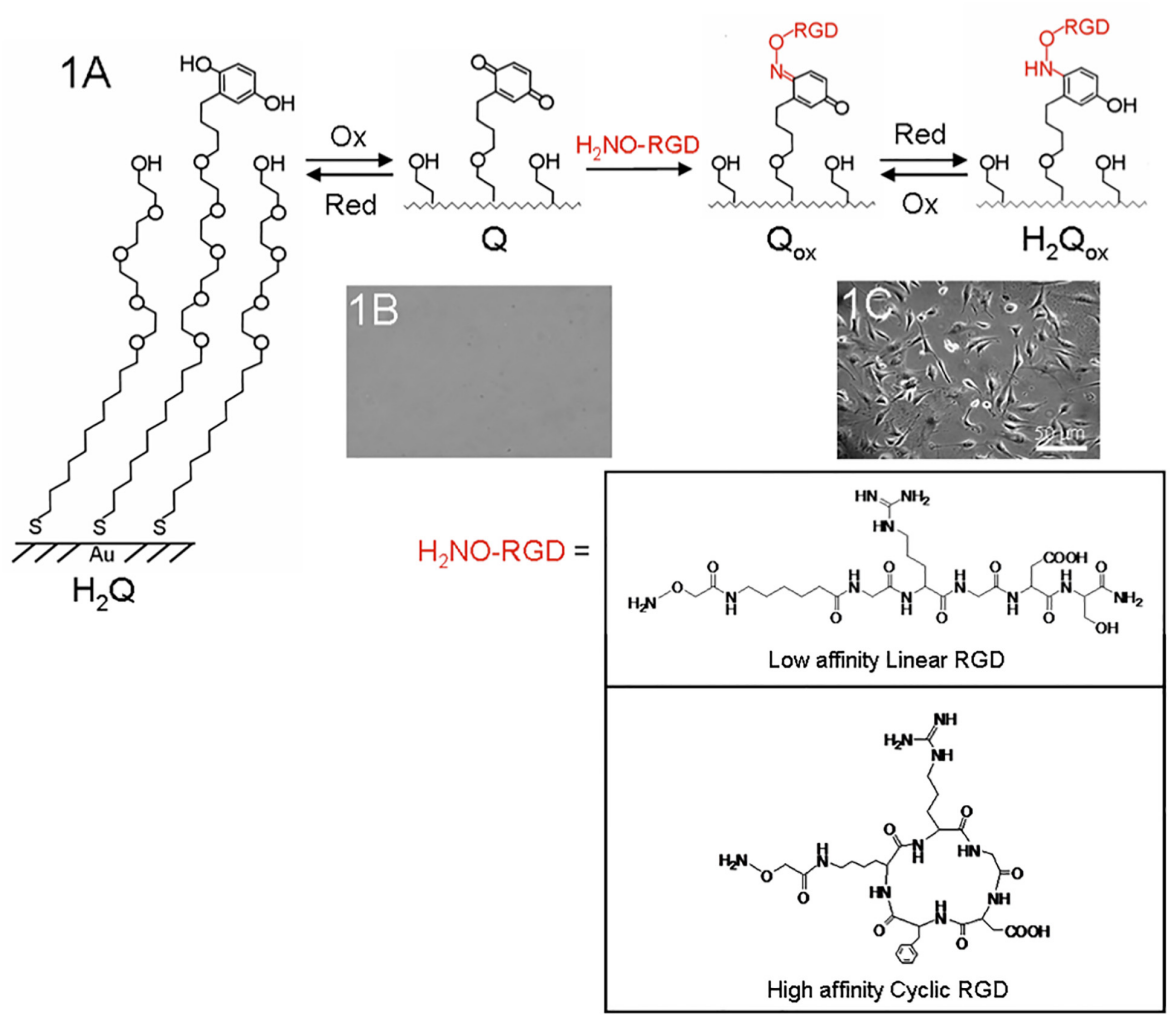
A chemical strategy to generate dynamic surfaces that can selectively turn on the immobilization of different affinity ligands (linear and cyclic RGD) for cell adhesion and growth. (A) A surface presenting a mixed monolayer of tetra(ethylene glycol) groups and redox active hydroquinone groups (H_**2**_Q) is oxidized to the corresponding quinone (Q) by applying an electrochemical potential to the underlying gold. Reaction of the quinone monolayer with soluble RGD-oxyamine at physiological conditions generated the peptide oxime conjugate on the surface. The oxime conjugate Q_**ox**_ is also electroactive and can undergo a reversible redox process resulting in the H_**2**_Q_**ox**_. The chemical structure of RGD-oxyamine is shown below. (B) The quinone monolayer, Q, is inert to the non-specific attachment of cells. (C) Addition of 3T3-Swiss albino fibroblasts to the RGD immobilized oxime surfaces resulted in cell attachment and proliferation.

Our approach for the dynamic substrate is based on an electrically switchable self-assembled monolayer (SAM) that presents redox active hydroquinone groups (Fig 1A). The substrate is essentially a working electrode that can oxidize and reduce the surface-bound molecules upon an applied potential. A mild electrochemical pulse readily converts the hydroquinone monolayer to the corresponding reactive quinone. The reversible redox coupling between the hydroquinone and the quinone is stable and can be characterized by cyclic voltammetry [17–18]. We have previously demonstrated that the resulting quinone monolayer can react rapidly and selectively with soluble oxyamine groups to form a chemically stable oxime conjugate on the surface [19]. By synthetically tethering ligands to the oxyamine, this methodology can potentially immobilize a variety of ligands such as peptides, carbohydrates, and other biomolecules to the surface. Furthermore, because the oxime conjugate undergoes redox coupling at different potentials from the quinone, the product possesses diagnostic waves that can be characterized electrochemically by cyclic voltammetry [19–21]. This feature permits the quantitative determination of yield and therefore density of ligand immobilized on the surface in situ. Most importantly, the highly selective coupling between the quinone and oxyamine prevents cross-reaction with other biomolecules including DNA, proteins, carbohydrates, and lipids. This coupling strategy therefore is ideal for bioconjugation in the presence of complex protein mixtures and in the presence of adhered cells in serum-containing media.

## Results and Discussion

### Ligand Immobilization for Cell Attachment

To determine whether the electroactive surface can support biospecific cell attachment *via* the oxyamine ligand immobilization strategy, we prepared mixed monolayer surfaces presenting 1% hydroquinone and 99% tetra(ethylene glycol) groups. The high density of background ethylene glycol groups within the monolayer has been shown to reduce non-specific protein adsorption [22]. This feature ensures that the cell-surface interaction is mediated only by the cell surface receptors and ligands on the surface. Electrochemical oxidation of the monolayer (750 mV for 10 s) converts the hydroquinone groups to the reactive quinones (Fig 1A). Addition of 3T3-Swiss albino fibroblasts to the resulting quinone monolayer showed no cell attachment to the surface (Fig 1B). This result demonstrates that the surface resists non-specific cell attachment and that electrochemical oxidation does not compromise the integrity of the monolayer. To facilitate cell adhesion we synthesized an oxyamine-tethered RGD peptide (structures in Fig 1A) and conjugated the peptide to the quinone monolayer (20 mM in PBS, 4 hrs). The RGD sequence is found within the central binding domain of several adhesive proteins present in extracellular matrix and is known to facilitate adhesion, migration and proliferation of cells on a variety of substrates and polymers *via* interaction with cell surface integrin receptors [23–26]. Addition of fibroblasts resulted in attachment and proliferation on the RGD-immobilized surface (Fig 1C). To show the attachment was specific we added soluble RGD peptide (final concentration of 1 mM, 1 hr) as a competitive inhibitor of the surface-bound RGD and the adherent fibroblasts detached from the surface (data not shown). Furthermore, we immobilized a scrambled peptide (GRD-oxyamine) to the quinone monolayer and observed no attachment of cells to the surface (data not shown). These results confirm that cell attachment to the monolayer surface was mediated only by the immobilized RGD ligands.

### Determination of Density of the Immobilized Ligands

To demonstrate the use of cyclic voltammetry to determine the density of immobilized ligands, we prepared monolayer surfaces presenting 50% hydroquinone and 50% tetra(ethylene glycol) groups (1:1 ratio on surface). S1 Fig shows the cyclic voltammogram corresponding to the hydroquinone-quinone redox coupling in 1M HClO_4_. Hydroquinone oxidizes at 540 mV and quinone reduces at 320 mV. Integration of the area under the reductive wave gave a density of 2.3 x 10^-10^ mol/cm^2^ for the quinone monolayer. Electrochemical oxidation, followed by immobilization of RGD-oxyamine (20 mM in PBS, 4 hrs) resulted in the oxime conjugate on the surface. Characterization of the monolayer by cyclic voltammetry shows diagnostic peaks that correspond to the oxidation (620 mV) and reduction (480 mV) of the oxime conjugate. Integration of the area under the oxime reductive peak gave a density consistent with the complete conversion of the quinone monolayers to the RGD oxime conjugate. The monolayers on gold are stable within the range of applied electrochemical potential and thus repeated redox scanning for several hours produce indistinguishable cyclic voltammograms. The ability of cyclic voltammetry to distinguish the oxime product from the initial hydroquinone-quinone redox couple enables the quantification of the amount of ligand molecules installed on the surface. From cyclic voltammogram data we found the pseudo-first order rate constant for immobilization to be approximately the same for both linear and cyclic RGD for surfaces ranging in quinone density from 50% to 2% as determined by electrochemistry and approximately 0.040 min^-1^ [19].

### Dynamic Surfaces for Biospecific Cell Migration

**Fig 2:**
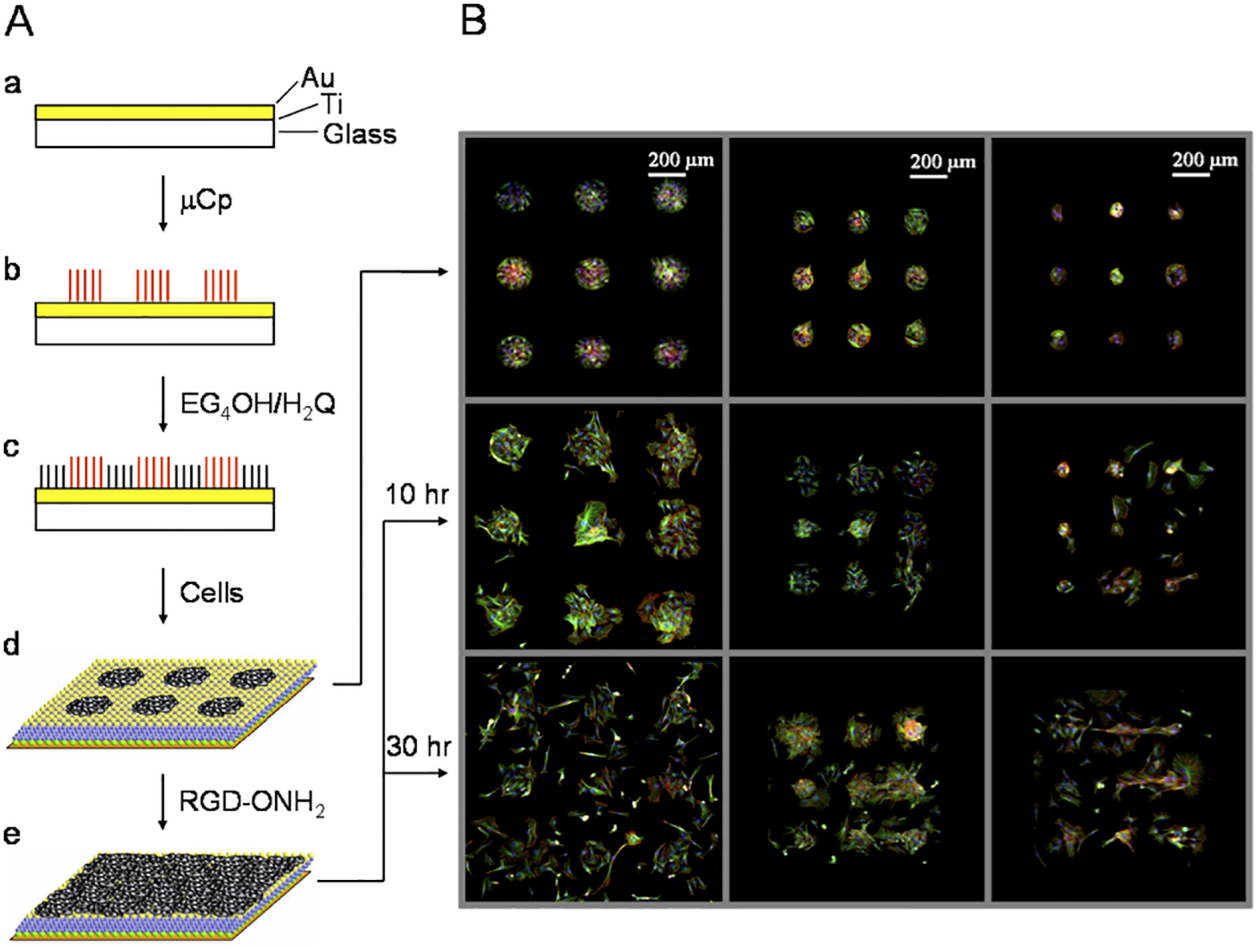
Example of a dynamic substrate for spatial and temporal control of cell migration and growth. (A) (a) Surfaces compatible with cell imaging were prepared by evaporating titanium (3 nm) and then gold (12 nm) onto glass coverslips. (b) Microcontact printing, a soft lithography technique, was used to pattern hydrophobic islands of hexadecanethiol. (c) The remaining bare gold regions were filled with a mixed monolayer of tetra(ethylene glycol) (EG_**4**_OH) and hydroquinone (H_**2**_Q) terminated alkanethiol (99:1 in ratio). (d) The patterned hydrophobic area was adsorbed by fibronectin and fibroblasts attached, proliferated and remained confined to the patterned regions. (e) Application of oxidative potential to the monolayer and subsequent addition of RGD-oxyamine (20 mM for 2 hours) (RGD-ONH_**2**_) resulted in the rapid immobilization of peptide and hence the migration and proliferation of cells off of the circular islands. (B) Representative figures from fluorescence microscopy over the period of time with three different sizes of circular patterns. The diameters of the pattern are 160 μm, 110 μm, and 60 μm, respectively. In all three sizes of the patterns, upon ligand presentation cells gradually migrated out and broke down the patterns completely within 30 hrs after RGD immobilization. Color: green, tubulin; red, actin filaments; blue, nuclei.

We next applied this methodology to generate dynamic model substrates for studies of cell migration. We used microcontact printing to first pattern hexadecanethiolate monolayers on the surface and backfilled the remaining bare gold regions with a mixed monolayer of 1% hydroquinone and 99% tetra(ethylene glycol) groups (Fig 2A). The hydrophobic patterns were then adsorbed with fibronectin (0.1 mg/mL in PBS, 2 hrs) to enhance cell attachment. Addition of fibroblasts to the surface resulted in cell attachment only to the microcontact printed regions. Observation by live-cell microscopy showed that while most cells are quiescent within the patterns, the cells near the edge of the patterns are highly dynamic (S1 Movie). The patterned cells constantly sample the surface microenvironment outside the pattern *via* filopodia and lamellipodia protrusions and membrane ruffling. Electrochemical activation converted the hydroquinone monolayer to the quinone, followed by immobilization of linear RGD-oxyamine (20 mM in serum-free medium, 2 hrs) to install the peptide ligands to the monolayer. The short electrochemical pulse applied (750 mV, 5 seconds) to oxidize the hydroquinone groups and the addition of the soluble oxyamine-functionalized RGD peptide do not affect cell viability. The surface microenvironment surrounding the cell patterns is switched from being inert to adhesive with surface-immobilized RGD ligands. As a result, cells confined within the confluent patterned region initiated migration in response to the changes in the surface microenvironment.

Fig 2B shows representative fluorescent images for ligand-mediated migration of patterned cells over time on three different sizes of circular pattern (160 μm, 110 μm, and 60 μm in diameter). After 2–3 hours of linear RGD immobilization, cells begin to protrude and move beyond the edge of the pattern, and after 10 hours the cells have moved a significant distance away from the initial circular patterns. Despite the different sizes of the initial patterns, all the cells continued to migrate and after 30 hours of RGD immobilization it was difficult to define the original pattern. As a control, the exact same substrates without the RGD immobilizing reaction were prepared. There was no migration and the cells stayed confined within the circular patterns even after 48 hours in the incubator (S2 Fig). As a further control to show the migration was due to the biospecific interaction between integrin receptors on the cell and the newly immobilized RGD ligands, soluble RGD was added into the cell medium (final concentration of 0.7 mM for 1 hr) and the cells detached from the RGD presenting regions but not from the hydrophobic fibronectin islands (data not shown). When a scrambled peptide (GRD-oxyamine) was immobilized under the same conditions (this peptide is not a ligand for integrin receptors) the cells were observed over 48 hours not to migrate from the patterns (S2B Fig). Therefore the dynamic presentation of ligands on the surface in the presence of the cells functioned as a molecular switch to turn on cell migration.

Unlike many conventional migration assays including wound healing assays [27, 28], Boyden chamber [29], and other methods [30–33] where cells migrate on ill-defined substrates, our dynamic substrate approach is mediated by the underlying surface chemistry where the ligand presentation, density, activity, and composition is defined at the molecular level. These features allow for the proper control and interpretation of cell behavior on tailored biospecific ligand-receptor mediated surfaces. Furthermore, because these substrates modulate the surface property in the presence of attached cell culture, it is possible to monitor cell behavior in response to various changes in the surface microenvironment in real-time (S2 Movie).

### The Role of Cell Population and Pattern Shape on Cell Migration and Growth

**Fig 3:**
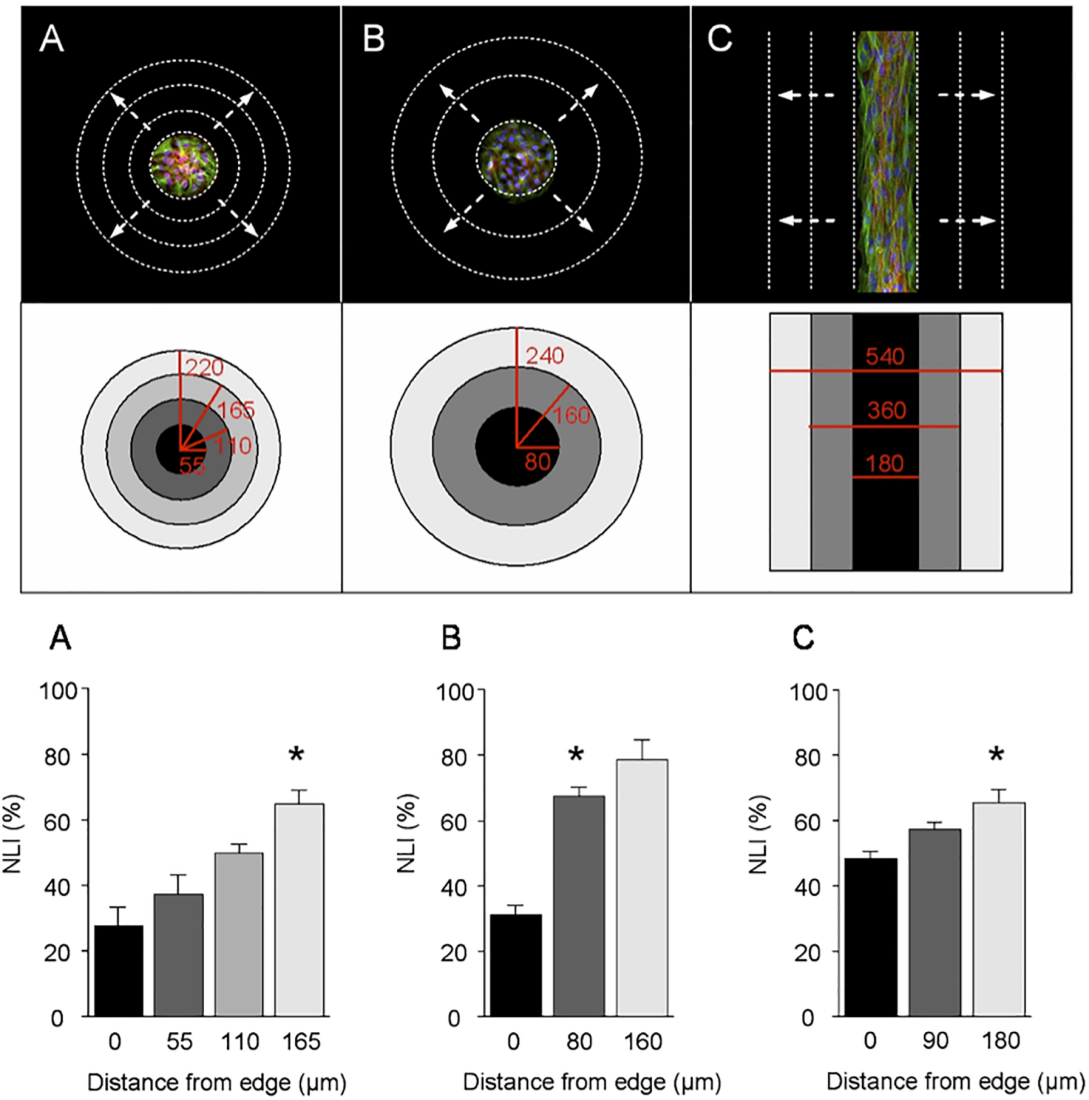
Example of cell migration and growth dependence on geometry and population of cells. On dynamic substrates fibroblasts were patterned on various sizes of circles and lines. Cells proliferated to fill the patterns at which time they exited the cell cycle and became quiescent (top panel shows that the monolayer of cells is confined to the patterned regions before migration is activated. Color: green, tubulin; red, actin filaments; blue, nuclei). Addition of soluble linear RGD-oxyamine (20 mM for 2 hours) installed the peptide at the density of 1% onto the surface. Fibroblasts were allowed to migrate off the patterns for 20 hrs in the incubator and BrdU was added in the culture medium to pulse-label the nuclei. By visualizing whole nuclei and BrdU-labeled nuclei, nuclear labeling index (NLI) was determined. NLI (%) is the ratio of newly synthesized nuclei to the whole nuclei. Cells were grouped by migrating distance from the initial pattern as shown in different colors of regions in the middle panel. The central area in black color is where the original pattern is and the NLIs in those regions are shown as the first bars in black color in each bar graph at the bottom. The colors of bar graphs correspond to the regions in the middle panel where number of nuclei was counted. (A) Cells were patterned in small circles that have low cell population (radius 55 μm) (B) Cells were patterned in large circles that have higher cell population (radius 80 μm) (C) Cells were patterned in a line with width of 180 μm. Regardless of pattern size and geometry there was a universal tendency where cells that migrated farther showed higher activity in synthesizing new DNA and therefore growth. In details this NLI measurement according to migration distances reflect delicate influences of cell-cell interaction and pattern geometry and they are described in the text with examples of *-noted bar graphs. Data are collected from 6 to 8 separate substrate chips each in (A), (B) and (C). Data are mean ± SEM.

We next determined how the number of cells and initial pattern shape affect motility and growth on the dynamic substrates. We used a nuclear labeling index (NLI) assay, which measures the amount of DNA synthesis occurring within adhered cells undergoing growth, to compare the role of pattern size and geometry on cell migration and cell growth. Dynamic substrates were prepared as described above and upon activation of the surface by immobilizing linear RGD, cells were allowed to migrate from the patterns for 20 hours and then 5-bromo-2’-deoxyuridine (BrdU, a thymidine analog that incorporates into newly synthesized DNA) was added in the cell culture medium (10 μM) for one hour to allow pulse-labeling of DNA. The cells were then fixed and the BrdU-labeled nuclei were detected *via* anti-bromodeoxyuridine and a Cy2-conjugated secondary antibody. Whole cell nuclei were also stained with DAPI and the results were visualized with fluorescence microscopy. To interpret the migration distance as a function of initial pattern geometry and size of the patterns, we defined areas by drawing circles or lines proportionally to the initial pattern radius or width to keep track of the cells according to the distance migrated (Fig 3 top and middle panel). The total number of nuclei and BrdU-labeled nuclei were counted in each area. The percentage of the newly synthesized nuclei (DNA) was calculated to give a NLI (ratio of BrdU-labeled nuclei to total nuclei). The bar graphs in Fig 3 show the NLI according to the migration distance. For the analysis, we assumed that cells initiated migration from the boundary edge of the pattern and the cells found within the interior of the patterned area had no migration upon surface activation. In the bar graphs of Fig 3A–3C, the first sets of data (distance 0 μm) represent the NLI of the cells within the initial pattern. These cells are considered as having no migration, and therefore their NLI is set as the basal level of new DNA synthesis in non-migratory, contact inhibited quiescent cells. The bar colors of the data set for each distance traversed corresponds to the regional zones of the same color in the pattern diagram above. Fig 3A and 3B compare the role of cell population in cell migration and growth by analyzing the NLI for small and large size of circular patterns that restrict the initial population of the cells. We also compare the effect of pattern geometry (circle versus straight line) on cell migration and growth (Fig 3C).

The comparison of NLI in relation to migration distance is a measurement to visualize the relative balance between migration and growth activity. We found that regardless of pattern size or shape the cells that migrated farther from the initial pattern had higher rate of new DNA synthesis (higher NLI). The cells that migrated the farthest were found to be the cells initially located at the edge of the pattern. Upon surface activation, these cells have greater access to free space that supports adhesion and migration and therefore are able to *turn on* their cell growth program before the cells originally located near the crowded center of the pattern do. These results may be also influenced by cell-cell interactions during cell movement. To control the cell number therefore population of cells we generated different size of patterns to determine how initial cell population influences subsequent cell migration and growth after surface activation. Interestingly, we observed that cells within the small and large circular patterns had similar basal levels of DNA synthesizing activity (27.5% and 31.2%, respectively) (Fig 3A and 3B). After surface activation, the cells which migrated approximately 165 μm (off small) and 80 μm (off large) circular patterns at the time point of 20 hours had similar levels of NLI (see the *-noted bar graphs with NLI of approximately 65%). These results show that for the same time period and growth rate (i.e. same NLI) cells from the small pattern (less cell population) migrated farther than cells from the larger population pattern. This result implies that there may be less cell-cell interactions in the smaller population patterns after surface activation and therefore the cells may migrate with less constraint where the dominating factor for migration is the number of cell-ligand interactions rather than the number of cell-cell interactions. Essentially, cells on the smaller pattern migrate farther because there are fewer cells on the small pattern to begin with and therefore have less initial cell-cell interactions on the small pattern. Cells on the larger pattern migrate less because there are more cells and therefore have to overcome more cell-cell interactions before they can migrate. The number of cells on the pattern influence how well/fast they migrate from the pattern after being released (allowed) to migrate.

For the cells initially confined within line patterns, we also observed that the farther the cells migrate the higher the growth rates (Fig 3C). We found that the basal level of DNA synthesis within the line patterns was much higher (48.4%) than for the circular patterns. Interestingly, the comparison between circular and line patterns (Fig 3A and 3C) showed a different trend for migration and growth. Within 20 hours, cells with approximately NLI 65% were found to migrate a distance of 180 μm from the large population line patterns which is similar to the distance cells travelled from the small population circular patterns. Although the cells migrate approximately the same distance and have similar NLI after 20 hours the cells on the line patterns took longer to initiate migration. For example, many cells on circular patterns have already migrated a significant distance within 10 hours after RGD immobilization on the surface while most cells on line patterns are not observed to migrate. This indicates the degree of curvature of the pattern where cells are originally confined can have a dramatic impact on the ability of cells to initiate migration and growth. On the straight line pattern the cells are densely packed parallel to the edge of the pattern and upon surface activation the cells at the edge must move out perpendicular to their alignment. This may require a massive rearrangement of the cell cytoskeleton and therefore it takes much longer to initiate migration compared to the cells on small circular patterns that are not restricted by geometry. However, after moving beyond the line patterns the cells are able to migrate with limited cell-cell interactions compared to the circular patterns where several cells are joined during migration. Therefore the comparable migration distance with the similar level of NLI in Fig 3A and 3C (*-noted bar graphs) appears to be influenced more significantly by the initial geometry. Overall the NLI measurements show that the actively migrating cells generally have higher growth rate and there is a delicate interplay of cell-cell interactions, cell-substrate interactions and initial geometry in dictating subsequent migration and proliferation.

### The Role of Integrin Composition on Cell Migration

We also investigated the role of integrin composition on cell migration and growth on the dynamic surfaces. We compared the rate of migration and growth for several Chinese Hamster Ovary (CHO) cell lines where the cytoplasmic α5 subunit of the α5β1 integrin, a fibronectin receptor, has been either deleted or truncated. Three mutant CHO cell lines were examined: B2a27 expresses a full-length of human α5 subunit with 27 amino acids in the cytoplasmic domain; B2a10 expresses an α5 with a 17 amino acid cytoplasmic truncation; and B2 expresses only the β1 subunit where the α5 is knocked down. In previous research Bauer *et al*. used these cell lines to examine the role of α5 cytoplasmic domain in cell adhesion, cell motility and cytoskeletal organization (*34*). On fibronectin-coated surface they found B2a27 and B2a10 displayed similar adhesion, motility and actin organization to wild type CHO cells while the nontransfected B2 cells showed no migration and no actin reorganization.

**Fig 4:**
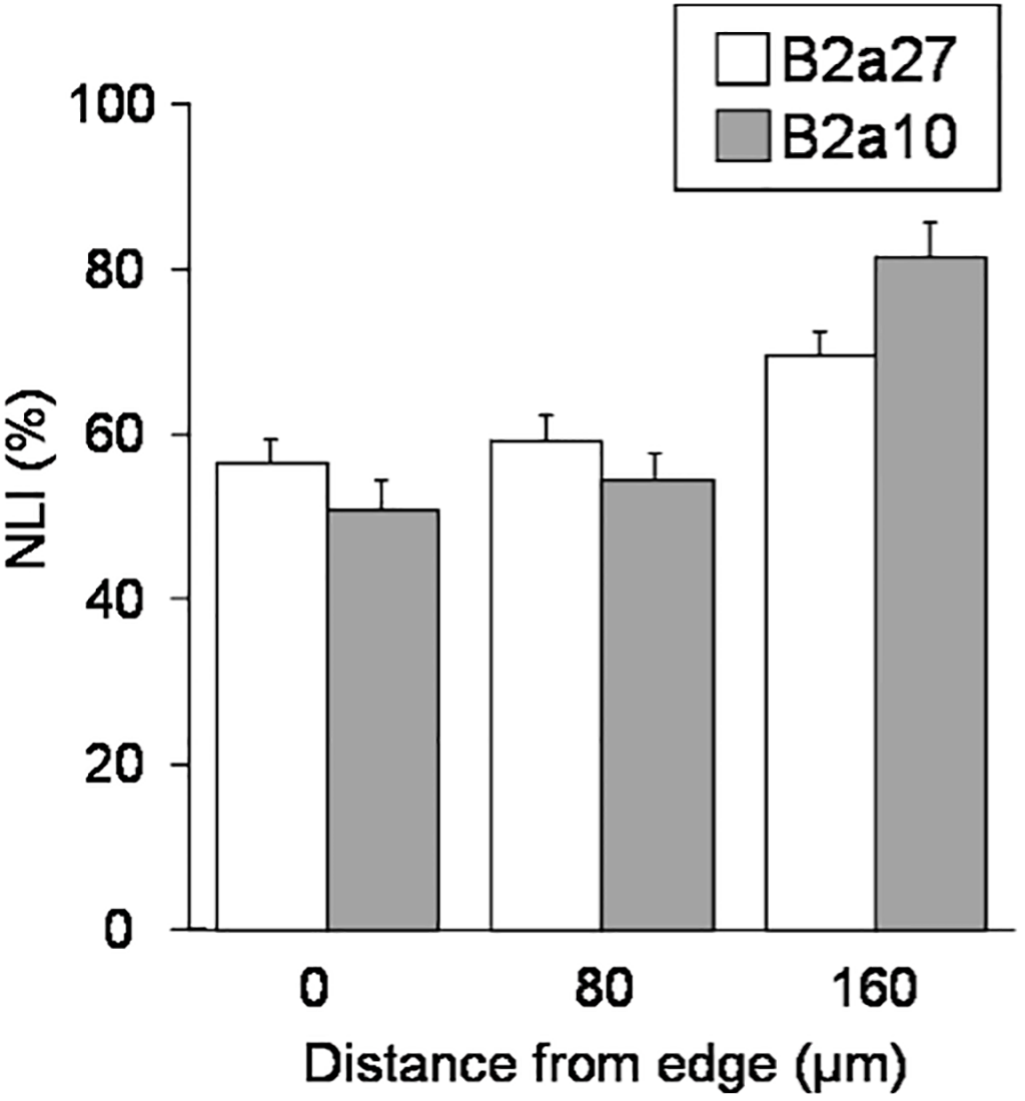
Growth versus migrating distance for integrin α subunit mutants of CHO cells on dynamic surfaces. B2a27 represents CHO cells with a full-length human α5 subunit with 27 amino acids in the cytoplasmic domain, B2a10 is a mutant with a 17 amino acid cytoplasmic truncation on α5 chain. With using these CHO cell lines, the experimental conditions and procedure were identical as in Fig 3B. Both cells had similar level of NLI along the migration distance to mouse fibroblasts (Compare with Fig 3B). The B2 mutants have a α5 knockdown and did not migrate from the patterns upon activation. Data are mean ± SEM and more than 6 separate substrate chips for each cell line were used for the analysis in the same procedure as in Fig 3.

To test whether these mutant cells behave in a similar manner on our molecularly defined surface, the same experimental procedure as in Fig 3B was performed with the three CHO cell lines. We found that both B2a27 and B2a10 cells patterned on 160 μm diameter circles migrated and proliferated to a similar extent as the 3T3-Swiss albino fibroblasts did upon activation on 1% linear RGD-immobilized surface (Fig 4). In contrast, although nontransfected B2 cells adhered to the initial pattern, they did not migrate when the surface was activated with linear RGD ligands. Thus, the truncated α5 cytoplasmic domain is not essential for cell motility but the extracellular domain and the cytoplasmic domain adjacent to the membrane may have important functions in cell migration. These cell motility and growth data is consistent with the results obtained from the fibronectin-coated surfaces [34]. Taken together, these results demonstrate dynamic surfaces are well-suited for evaluating the dependence of cell growth and migration on integrin composition and expression levels.

### On Non-Dynamic Surfaces Cell Migration Rate Depends on Ligand Affinity and Density

**Fig 5:**
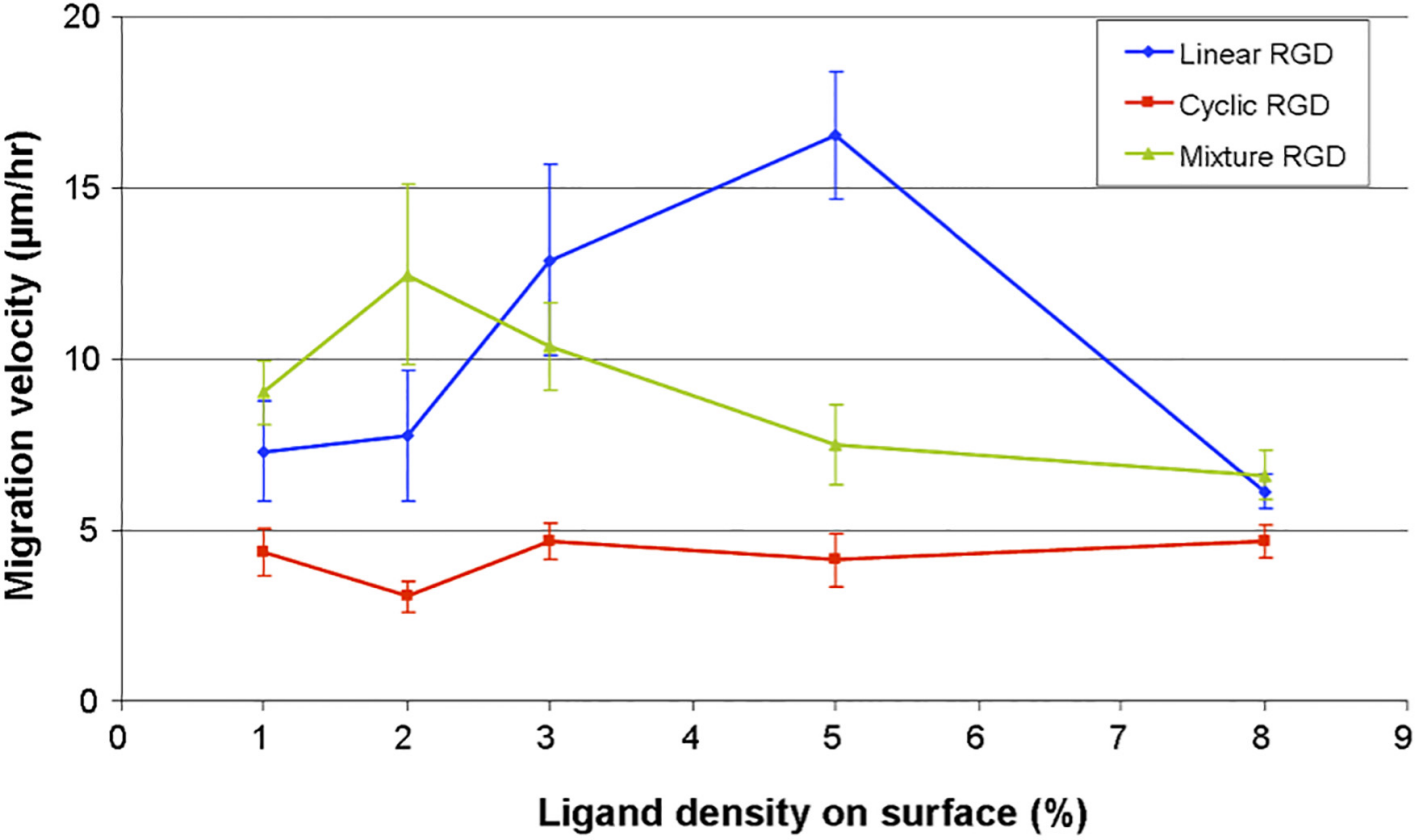
Plot of cell migration velocity versus surface density of linear, cyclic and mixture RGD peptide on non-dynamic surfaces. For cyclic RGD surfaces (red line) the cell velocity does not change significantly as the peptide concentration increases. For linear RGD peptide surfaces (blue line) there is a biphasic behavior where cell velocity increases as peptide density reaches from 1% to 5% and then decreases at higher densities of peptide. For mixture peptide surfaces (green line) there is also a biphasic behavior but the maximum velocity occurs at lower peptide density (2%). The migration velocity is slower for cyclic RGD surfaces for all densities than the other two peptide surfaces. Among the lowest density of peptides (1%) the mixture surface renders the highest velocity. At higher peptide densities than 8% non-specific interactions dominate and the cell migration velocities uniformly increase. The experiment was repeated twice for each surface. Seven to ten individual cells from each experiment were tracked for 30 hrs to give mean velocity. Data are mean ± SEM.

To understand the role of ligand density and affinity in directing cell migration we performed a series of studies where cell migration rates were monitored on surfaces presenting different densities and affinities of RGD ligands. Integrin receptors are known to have higher affinity to cyclic RGD than linear RGD (IC_50_[cyclic RGD] / IC_50_[linear RGD] ≈ 10^-3^) [35–36]. To modulate the ligand affinity on our surface, we used linear RGD, cyclic RGD, and a mixture of linear and cyclic RGD (1:1 ratio). For these studies SAMs were prepared as mixed monolayers presenting hydroquinone (from 1% to 10%) and tetra(ethylene glycol) (99% to 90%) groups on gold. Upon electrochemical oxidation to quinone, linear RGD, cyclic RGD or mixture of linear and cyclic RGD peptides were immobilized. The completion of coupling reaction was determined by cyclic voltammetry. Each substrate was rinsed with water and 3T3-Swiss albino cells were then seeded to determine migration velocity as a function of peptide density. We term this type of substrates as a “non-dynamic surface” since there is no patterned region of cells and the entire surface is composed of peptide ligands prior to cell seeding. Time-lapse images were recorded and the movement of the cells was tracked. Fig 5 compares cell velocity on the surfaces with different density and ligand affinity. For the non-dynamic high affinity cyclic RGD surfaces, the attached cells rarely migrate and there is no substantial difference in cell migration rate on 1 to 8% ligand density. This reflects that the polyvalent adhesive interaction between integrin receptors and cyclic RGD ligands is too strong even at low densities to allow significant cell motility. However for low affinity linear RGD surfaces, we observed a biphasic behavior where cell migration velocities increased up to 5% ligand density and then decreased from 6% to 8%. The biphasic cell migration rate data show that at low ligand density (1–5%) cells are able to attach and release integrin receptors from the surface and migrate with increasing velocity but at higher ligand densities (> 5%) the adhesiveness of the surface overcomes the ability for the integrin receptors to detach and therefore the migration is not effective. Interestingly, for the mixture RGD surface (linear and cyclic RGD are in equal ratio) the migration rates were faster at lower density and exhibited an earlier biphasic behavior than for the linear RGD surfaces. From our studies a mixture RGD surface allows the greatest cell motility at 1–2% ligand density and at higher densities the effect of the cyclic RGD predominates and causes motility to significantly decrease. Why cells migrate in higher velocity on the mixture RGD surface than on the linear RGD surface at < 2% ligand density is intriguing. It may be because cells possess the ability to modulate the combinatorial variability of the affinity states of the integrin receptors for complex ligands. Previous work has shown a biphasic migration rate on fibronectin-coated surfaces depending on the concentration of the fibronectin solution used [37]. However, these surfaces are unable to define the ligand orientation and the actual ligand density on the surface because they rely on non-specific fibronectin adsorption. Because our surface is based on molecularly well-defined ligand coupling and presentation, it is ideal to study the influence of ligand concentration and affinity on cell motility. The results presented here are significant because there have been no studies of cell motility on different affinity and mixed affinity peptide presenting surfaces.

### Determining Cell Migration Memory on Dynamic Surfaces—The Dynamic Presentation of Ligands Alters Cell Migration Rates

**Fig 6:**
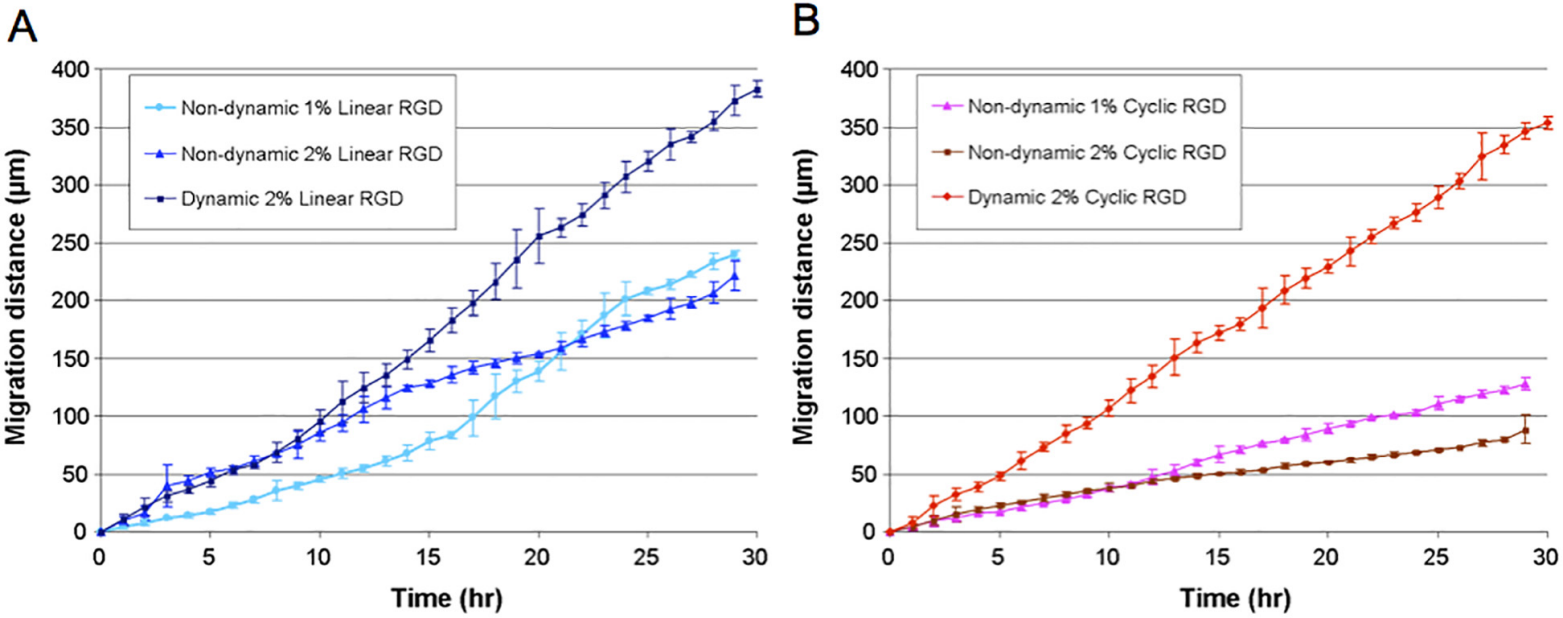
Comparison and evaluation of cell migration memory on dynamic and non-dynamic surfaces. The total distance cells migrated on dynamic or non-dynamic surfaces was plotted over 30 hrs. For dynamic surfaces, peptide was immobilized in the presence of cells on 160 μm diameter circular patterns and their migration was captured by time-lapse microscopy. The movement of cells every hour was tracked and summed up to get total distance cells migrated by that time. For non-dynamic surfaces, cells were seeded on the peptide-immobilized SAM surfaces. The peptide density was controlled and identical on dynamic and on non-dynamic surfaces. Cell images on non-dynamic surfaces were also captured by time-lapse microscopy and the movement was tracked in the same manner as dynamic surfaces. A. Cell migration on linear RGD surface. The average migration velocities were obtained from the slope of best fit line and they are 12.3 μm/hr (dynamic 2%); 7.3 μm/hr (non-dynamic 1%); 7.8 μm/hr (non-dynamic 2%). B. Cell migration on cyclic RGD surface. The average migration velocities are 11.6 μm/hr (dynamic 2%); 4.4 μm/hr (non-dynamic 1%); 3.1 μm/hr (non-dynamic 2%). Overall, cells on the dynamic surfaces migrated much faster than cells on the non-dynamic surfaces regardless of ligand affinity. The cells seem to have the ability to modulate their migratory behavior depending on their initial location and environment that overcomes the surface composition. For each surface the experiment was repeated 6 times. The movement of a total of 10 to 12 cells on each surface was tracked and data are mean ± SEM.

For metastatic cells the ability to migrate with different behavior and rates when originating from a tumor is of major interest in cancer biology. Although metastasis from a primary tumor is generally considered as a result of the complex interplay of random mutations, pre-existing genetic background and the local microenvironment, some types of tumors are metastasized by nonrandom process [38–39]. In this case, the microenvironment within and outside the primary tumors provide a selective path for metastasis. To explore the role of the dynamic microenvironment on cell motility, we wanted to test a novel hypothesis—whether initial cell adhesion site and surroundings would influence subsequent migratory behavior of the cells or do cells have a migration memory that modulates their migration behavior after leaving the initial adhesion location. For this study, we employed the dynamic substrates to compare the rate of migration of cells from patterns onto regions presenting the same RGD ligand density and affinity as non-dynamic surfaces. If the initial location and environment affects cellular behavior, the dynamic and non-dynamic surfaces may induce different migratory behavior responses although the surface chemistry composition (ligand type and density) are identical. For this study we compared total migration distances over time and the plots are shown in Fig 6.

For the *non-dynamic surfaces*, the migration on cyclic RGD is much slower than on linear RGD as expected due to the much higher affinity of cyclic RGD for integrin receptors (Fig 5). However, we observed on *dynamic surfaces* cells migrate much faster (from the initial patterns) regardless of ligand affinity. The average velocity of cell migration is similar for linear and cyclic dynamic surfaces (12.3 μm/hr and 11.6 μm/hr respectively). The most striking result is for the dynamic and non-dynamic cyclic RGD surfaces. The cells migrated much faster on dynamic surfaces when released from the initial pattern presenting 2% cyclic RGD (S3 Movie) whereas on the non-dynamic 2% cyclic RGD surface the cells migrated much more slowly (Fig 6). This result clearly demonstrates that cells retain a *memory* of their initial pattern or state and when released from the pattern they are able to modulate their ability to migrate that overcomes the adhesiveness of the ligands presented.

**Fig 7:**
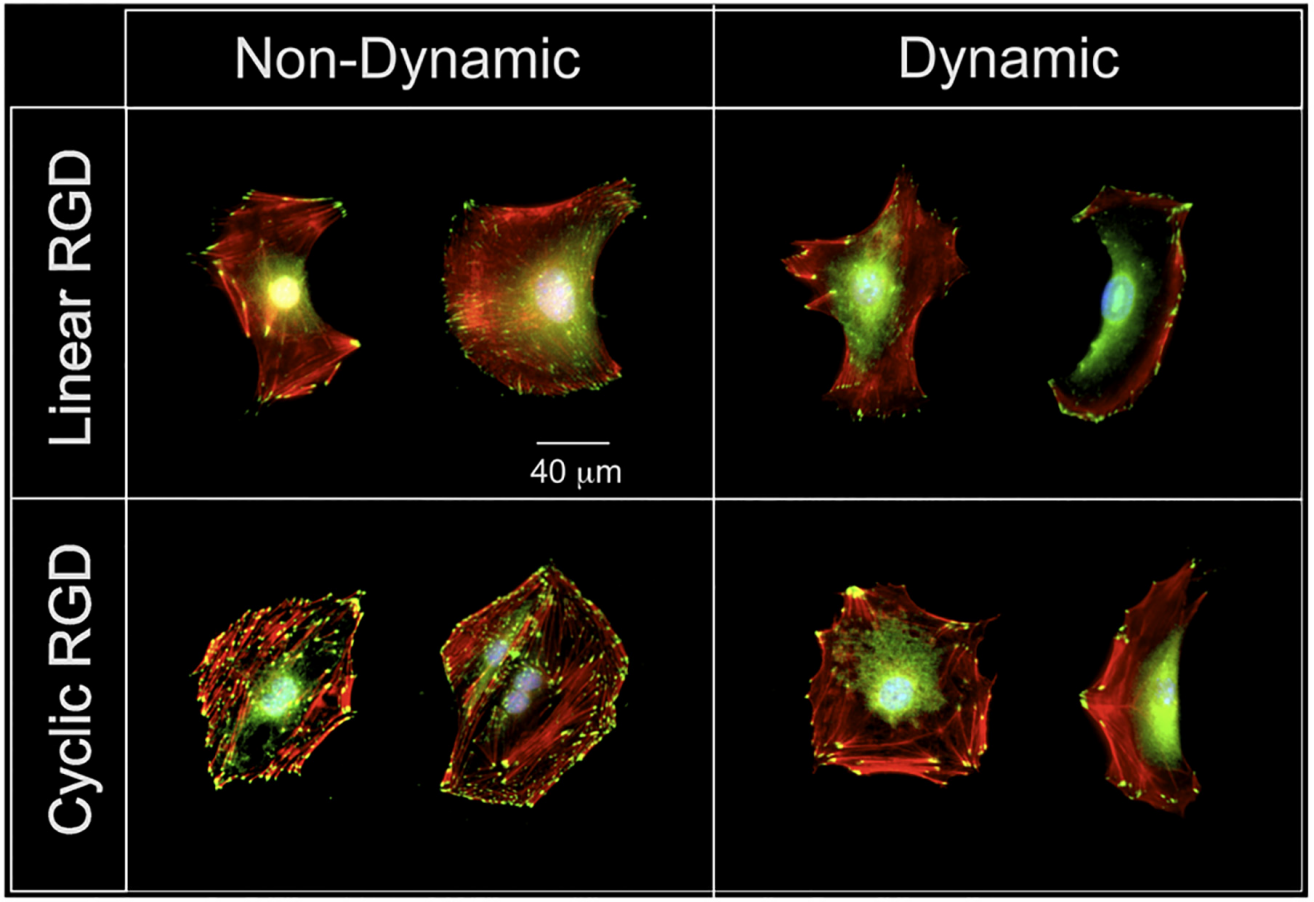
Comparison of fluorescent micrographs of fibroblasts with focal adhesion staining on dynamic and non-dynamic surfaces. (Left column) On non-dynamic surfaces, cells attached to linear RGD peptides have fewer numbers but more localized focal adhesions on the periphery of the cell comparing to the cells adhered to cyclic RGD surfaces where more focal adhesions are found throughout the cell body. (Right column) On dynamic surfaces, very similar number and distribution of focal adhesions are found within the cells for both linear and cyclic RGD surfaces after cells are allowed to move off the initial circular patterns. The striking difference is the comparison between focal adhesion staining of the cells on dynamic-cyclic and non-dynamic—cyclic RGD surfaces. Although the surface ligand composition is identical the cells chose different migratory behavior on the dynamic and non-dynamic cyclic RGD surfaces reflected in velocity and focal adhesion distribution. Cell-substratum interactions on non-dynamic cyclic RGD surface is dominated by the high affinity of the cyclic-RGD ligand and many stable focal adhesion complexes are distributed throughout the cell body. However, cells on dynamic cyclic RGD surface are highly motile and focal adhesion formation is more transient and localized only at the periphery. It is thought that cells have a memory of their initial state in which they were densely packed and contact inhibited and may influence their subsequent migratory behavior on the dynamic surfaces. Color: green, paxillin; red, actin; blue, nuclei.

To further investigate these results we examined the focal adhesion structures within cells. Focal adhesions are large, dynamic protein complexes through which the cytoskeleton of a cell is connected to the extracellular matrix (ECM) [5, 40]. The assembly and morphology of focal adhesions are critical in signal transduction during ligand mediated cell adhesion polarization and migration. The focal adhesion structure of adhered cells on the surfaces were analyzed by visualization with an anti-paxillin antibody. Paxillin is a protein found within focal adhesions and is a well characterized marker for these structures [41]. More motile cells are known to have fewer focal adhesions and they are found at the periphery of the cell, which enhances attachment and detachment from the surface during active migration. Fig 7 shows focal adhesion staining of representative cells migrating on dynamic and non-dynamic linear and cyclic RGD surfaces. Interestingly, cells on the higher affinity non-dynamic cyclic RGD surfaces (bottom left micrograph) have more and larger focal adhesions and therefore cells migrate intermittingly and slowly, but on the dynamic surface presenting cyclic RGD (lower right micrograph) the cells have focal adhesions only on the periphery and are very motile and look indistinguishable from cells on lower affinity RGD surfaces (top row). Since the cells are contact inhibited on the patterned islands we hypothesize that once they are allowed to migrate their motility program supersedes the affinity of the substrate in order for the cells to find more space to migrate and divide. We expected once the cells moved off the patterns onto high affinity cyclic RGD surfaces that the cells would immediately halt their migration due to the very high affinity of cyclic RGD to their cell surface integrin receptors. Instead, the cells migrated at a greater velocity and therefore the cells are somehow able to retain a memory of their initial pattern and are able to either down-regulate their integrin receptors or change the affinity state of the integrins to migrate. We also observed for longer periods (> 20 hrs) some cells divided and the resulting daughter cells continued to be very motile and had similar focal adhesion size and number as the parent cells. This result brings the issue of persistence of cell motility memory that is carried through cell division. We are currently investigating whether the integrin receptors remain in the high active state of migration (with selective integrin antibodies) which is carried through cell division to the daughter cells. We are also actively studying the role of cell-cell communication, integrin activity (regulation) state and key gene signaling levels in order to decipher the complex signaling that regulates this behavior. These results show that cells are able to modulate their migration behavior on a *molecularly* defined surface based on their initial position and may be important to understanding basic cell migration and the transition to metastasis for cancer cells from tumors.

### Whole Genome Microarrays Reveal Differential Gene Expression on Dynamic and Non-Dynamic Surfaces

To investigate how the cells are able to modulate their migratory behavior at the genomic level on different RGD-ligand affinity dynamic and non-dynamic surfaces, we incorporated whole genome microarray analysis with these molecularly tailored surfaces. We used this strategy to identify key genes that may be responsible for the cell migration memory mechanism that is employed to regulate cell behavior on different surface compositions.

**Fig 8:**
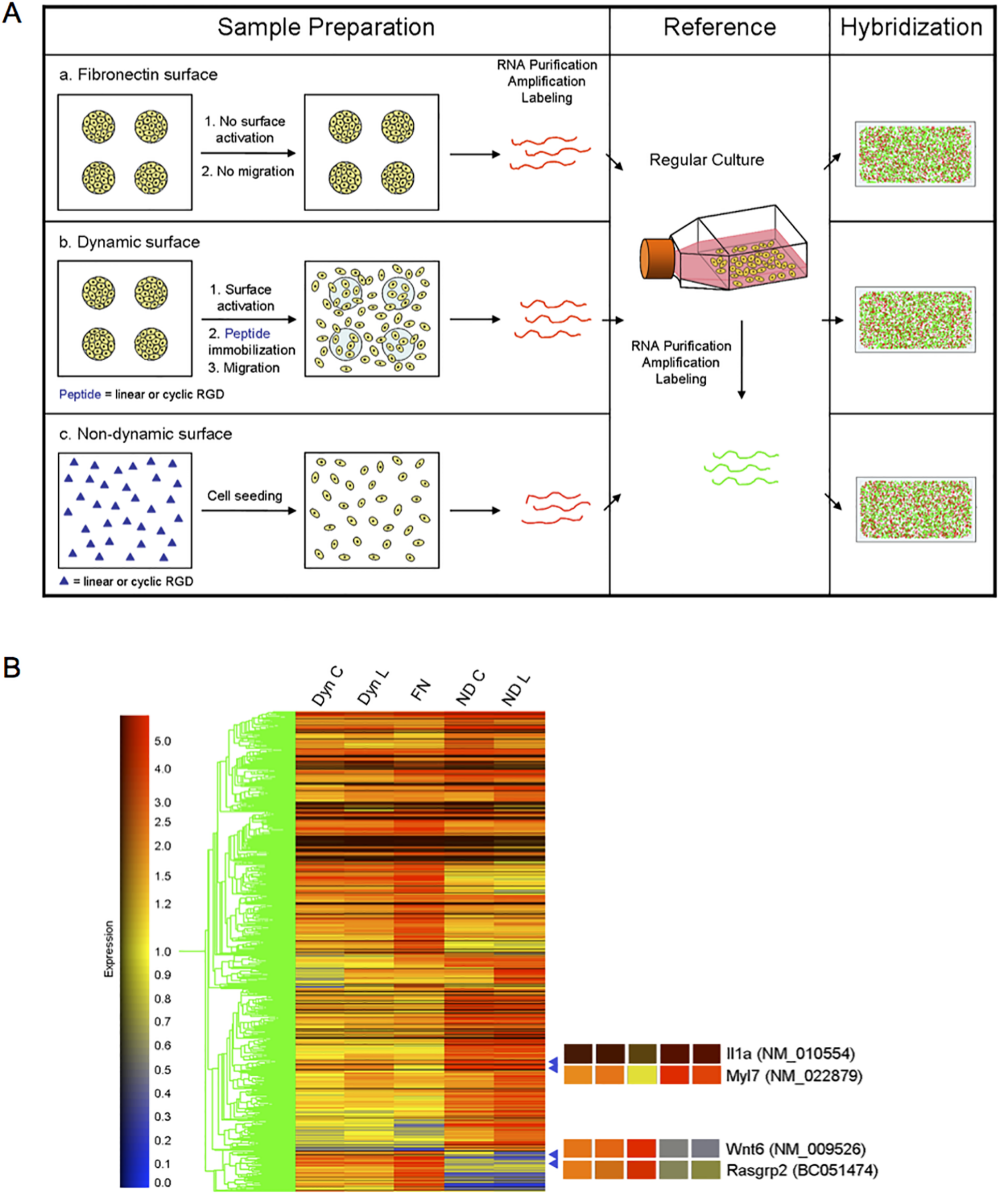
Comparison of gene expression by whole mouse genome microarray. (A) Schematic diagram of two-color whole genome microarray hybridization experiment. a. Fibronectin surface—microcontact printed area was adsorbed with fibronectin to enhance cell attachment. No RGD was added thus there was no cell migration; b. Dynamic surface—in the presence of the cells on the fibronectin patterns, RGD (linear or cyclic form) was immobilized. Cells were allowed to migrate for 24 hours in complete medium at 37°C, 5% CO_2_; c. Non-dynamic surface—linear or cyclic RGD was immobilized on the SAM surface *via* an interfacial oxime linkage. Cells were added onto the surface and incubated for 24 hours in the same condition as above. From each surface cells were collected by trypsinization and RNA was extracted. RNAs were amplified using random primers and labeled with Cy5. For the reference of hybridization experiment, cells grown in common culture flask were harvested. RNA was extracted and amplified in the same method and labeled with Cy3. Labeled nucleic acid samples (a, b and c) and reference were used in microarray hybridization. After scanning the microarray slides, the intensity of two colors in each array was normalized and analyzed. (B) Hierarchical cluster analysis of microarray data. The expression profiles of 1716 genes whose intensity differed 2.5-fold or more between the reference and at least one sample were chosen for hierarchical clustering analysis with Genespring software. The cluster image was created according to underlying similarities in patterns of gene expression. The column represents each sample group, in which the mean of four separate microarray data are shown (t-test p-value less than 0.05). The genes are listed in rows. The normalized expression level for each gene is coded by color. Red indicates high expression and blue indicates low expression in each sample. For example, Il1a, interleukin-1-α, is extremely up-regulated in non-dynamic surface samples but only mildly up-regulated in dynamic and fibronectin surface samples. Myl7, Myosin light polypeptide 7, is significantly highly expressed in dynamic and non-dynamic surface samples while it is down-regulated in fibronectin surface sample (gene accession number in parentheses). The expression of Wnt6 and Rasgrp2, wingless-related MMTV integration site 6 and Ras guanyl releasing protein 2 respectively, are up-regulated on dynamic surfaces and fibronectin surface samples while the non-dynamic surface samples have much lower expression. [Dyn C], dynamic surface with cyclic RGD; [Dyn L], dynamic surface with linear RGD; [FN], fibronectin patterned surface; [ND C], non-dynamic surface with cyclic RGD; [ND L], non-dynamic surface with linear RGD].

A total of five different groups of samples were compared and are illustrated in Fig 8. For fibronectin surfaces, fibroblasts were seeded onto fibronectin patterns to which they adhered and became contact inhibited. The cells and substrate were not exposed to RGD ligand and therefore cells were not able to migrate and remained confined to the patterns (Fig 8A, a). For dynamic surfaces, the substrates with patterned cells were activated electrochemically and RGD peptide immobilization was followed (Fig 8A, b). Either cyclic RGD or linear RGD was installed onto the surface. Upon recognizing the RGD ligand, cells initiated migration. For the non-dynamic surface, RGD peptide (cyclic or linear form) was coupled to the surface before adding cells (Fig 8A, c). For both dynamic and non-dynamic substrates, cells were incubated for 24 hours after migrating on immobilized RGD ligand surfaces. Cells on the fibronectin surface were also incubated for 24 hours. After this period of time, cells from each substrate were collected and RNA was extracted. Total RNA was purified, amplified and labeled with Cy5. As a reference, RNA was obtained from regularly cultured cells in tissue culture flasks and incorporated with Cy3 after purification and amplification. Each sample and reference were hybridized onto a whole mouse genome microarray. Each microarray analysis visualizes differential gene expression between reference and the sample. By normalizing the data, gene expression levels between sample groups can also be compared.

Cell numbers in regular culture were maintained below 70–80% confluency, hence these cells are regarded as proliferating and migrating at a normal rate. Cells on the fibronectin patterns are close packed and the resulting contact inhibition arrests both growth and migration. However, cells on the dynamic surface and non-dynamic surface are permitted to migrate freely on the RGD tailored surfaces, although how the cells gain access to the RGD ligands to initiate migration is different (see discussion of dynamic vs. non-dynamic surface for cell migration above). A cluster image created by GeneSpring software visualizes differential gene expression levels between samples (Fig 8B). The gene expression pattern profiles are strikingly different for dynamic, non-dynamic and fibronectin surfaces. The ligand affinity between the cyclic and linear form of RGD also had interesting gene expression profile differences between dynamic and non-dynamic surfaces. For example, Myl7 (Myosin, light polypeptide 7, regulatory) which functions in focal adhesions, tight junctions and regulation of actin cytoskeleton shows higher expression on dynamic surfaces than the reference. In non-dynamic surface conditions it is expressed at even higher levels while fibronectin surface conditions kept the expression level similar to that of reference. Wnt6 (Wingless-related MMTV integration site 6) which is important in the Wnt and hedgehog signaling pathways is up-regulated on the fibronectin surface and dynamic surface but is significantly down-regulated on non-dynamic surface conditions. Cells on both dynamic and non-dynamic surfaces that had been exposed to RGD peptide expressed high levels of IL1a (interleukin-1-α). IL1a is known to be produced as a response to infection or environmental change to stimulate growth and initiate an immune response. Interestingly the expression level is 3 to 4-fold higher on dynamic substrates, but cells that were directly added onto RGD surfaces expressed extremely high level (15 to 24-fold) of IL1a. Rasgrp2, Ras guanyl releasing protein 2, is a component in MAPK signaling pathway and it can activate Ras. It is up-regulated on dynamic and fibronectin surface samples but on non-dynamic surface samples it has only 60% the level of regular expression.

Genes that show distinguished patterns between sample surface groups are summarized in Table 1. The numbers in the table represent the fold change in expression level compared to the reference sample. Many genes are found to be related to cell signaling pathways which regulate proliferation, migration, development and apoptosis. Also several genes functioning in focal adhesion and cytoskeleton organization/biogenesis are regulated differently according to the surface conditions. The integrin subunits responsible for fibronectin binding (α5β1) are expressed at similar levels for dynamic surfaces compared to regular culture. They are slightly up-regulated on non-dynamic surfaces while down-regulated on the fibronectin surface. A few other integrin subunits show characteristic expression patterns between samples while most integrin subunits have little variation in expression level when compared to the reference. Whether the regulation of such integrin subunit numbers on the cell surface at the transcription level is directly linked to cell migration is not clear. Some extracellular matrix protein genes are regulated at different levels depending on the surface conditions and how the cells use them to modify the surface for subsequent migration is to be investigated. From the overall microarray data analysis several of the genes that have the greatest difference in expression levels have roles in certain key signaling pathways related to proliferation and migration. In particular, a few candidate genes in the Wnt and Hedgehog signaling pathway are currently under investigation for their specific role in cell motility and proliferation mechanisms that influence cell migration memory. This genomic approach coupled with proteomic analysis may reveal a signature map that regulates cell motility and cell migration memory. An extended gene list for each pathway category is shown in (S1 Table).

**Table 1:** Genes that show distinguished patterns between sample surface groups.

Category	*Dyn C	Dyn L	FN	ND C	ND L	Name	Symbol
Signaling	3.09	3.75	2.78	15.64	13.12	Wingless-related MMTV integration site 2	Wnt2
	2.51	2.90	5.77	0.50	0.48	Wingless-related MMTV integration site 6	Wnt6
	2.48	3.22	8.89	0.81	0.89	Axin2	Axin2
	2.75	2.57	3.00	0.67	0.77	Secreted frizzled-related protein 1	Sfrp1
	3.35	3.81	1.46	23.96	15.28	Interleukin 1 alpha	Il1a
	2.37	2.37	4.91	0.60	0.69	RAS, guanyl releasing protein 2	Rasgrp2
	2.42	3.52	5.68	1.37	1.11	Patched homolog 2	Ptch2
	1.31	1.81	1.17	15.94	14.69	Amphiregulin	Areg
Focal Adhesion/ Regulation of Actin	4.05	3.09	5.99	9.48	15.43	Von Willebrand factor homolog	Vwf
	3.09	3.09	3.62	13.86	10.71	NCK associated protein 1 like	Nckap1l
	1.54	0.75	2.55	0.28	0.23	Insulin-like growth factor 1	Igf1
	1.65	1.88	0.50	0.46	0.29	Actin, alpha 2, smooth muscle, aorta	Acta2
	2.03	2.50	0.90	5.60	4.09	Myosin, light polypeptide 7, regulatory	Myl7
Integrins	7.32	8.38	13.85	1.12	0.63	Integrin, alpha 11	Itga11
	1.50	1.92	1.64	5.39	7.78	Integrin alpha 2	Itga2
	1.04	0.98	0.56	1.80	2.85	Integrin alpha 5 (fibronectin receptor alpha)	Itga5
	1.24	1.02	0.62	1.25	1.57	Integrin beta 1 (fibronectin receptor beta)	Itgb1
Adhesion Molecules	1.34	1.54	3.02	0.64	0.48	Intercellular adhesion molecule	Icam1
	0.88	0.86	2.42	9.45	9.69	Cadherin EGF LAG seven-pass G-type receptor 1	Celsr1
	1.50	1.80	1.30	5.38	5.56	Cadherin 5	Cdh5
	1.53	1.63	0.98	12.77	16.94	Cadherin 6	Cdh6
	2.71	2.81	3.57	10.13	8.98	Junction adhesion molecule 2	Jam2
Extracellular Matrix	1.66	1.56	3.00	0.49	0.32	Procollagen, type II, alpha 1	Col2a1
	3.82	4.78	7.82	3.86	4.20	Procollagen, type XVIII, alpha 1	Col18a1
	6.63	5.24	7.51	1.23	0.98	Laminin, alpha 4	Lama4
	0.71	0.68	0.52	4.56	5.20	Laminin, beta 3	Lamb3
Growth	1.43	1.81	2.31	8.47	6.59	Fibroblast growth factor 21	Fgf21
	1.84	2.19	0.49	2.74	2.22	Connective tissue growth factor	Ctgf
	3.47	2.70	1.33	4.69	3.38	Gremlin 2 homolog, cysteine knot superfamily (Xenopus laevis)	Grem2
	0.66	0.84	0.87	3.81	3.65	Growth differentiation factor 9	Gdf9
Apoptosis	0.86	1.31	1.80	1.29	1.38	Caspase 9	Casp9
	0.52	0.47	1.13	1.23	1.69	Aldehyde dehydrogenase 1 family, member L1	Aldh1l1
	3.78	4.38	4.83	11.56	12.36	Aldo-keto reductase family 1, member C18	Akr1c18

*Symbols: Dyn C—Dynamic surface with cyclic RGD Dyn L—Dynamic surface with linear RGD FN—Fibronectin patterned surface ND C—Non-dynamic surface with cyclic RGD ND L—Non-dynamic surface with linear RGD

### Conclusion

A dynamic substrate has been generated and shown to have spatial and temporal control of cell behavior. The model substrate is based on an electroactive surface where patterned cells can be released upon mild electrochemical activation to install adhesive ligands which are required to initiate and support cell migration and growth. The use of the reversible hydroquinone-quinone system that is under external electrochemical control allows for the quantitative real-time change in surface properties in the presence of adhered cells. The chemoselective reaction between soluble oxyamine-tethered ligands and the quinone form of the redox couple is compatible with cell culture conditions and this synthetic flexibility allows for tailoring the surface with a variety of biomolecules and ligands. The use of cyclic voltammetry to distinguish between the diagnostic peaks of the product oxime and the initial hydroquinone-quinone peaks is essential for characterizing the extent of the interfacial reaction and therefore allows for the precise control of ligand density on the surface.

We have shown the dynamic surface has exquisite molecular control of the ligand-receptor interaction between cell and material and demonstrated the utility of this methodology by studying the interplay of cell population, ligand density, geometry and integrin composition on cell behavior in real-time. We have also shown for the first time a novel behavior of cell migration memory where cells are able to change their migration velocity and focal adhesion patterns depending on their initial position that supersedes the underlying surface chemistry. Furthermore, whole genome microarray analysis showed several genes regulating cell signaling, cytoskeleton organization and proliferation are expressed at significantly different levels depending on the spatio-temporal control of the surface composition. This genomic profiling analysis combined with proteomic data will further initiate studies to explore at the systems biology level the key components responsible for cell motility and cell migration memory behavior.

These dynamic surfaces potentially have the ability to modulate several ligands simultaneously to adhered cells and may also be used to control the spatial and temporal interactions between two or more different populations of attached cells for co-culture, multi-cell array and tissue engineering applications. Because of the synthetic flexibility of the methodology and the compatibility with high throughput and parallel assay technologies, library of peptides, carbohydrates and small molecules can be immobilized and screened for a variety of studies and applications [42–45]. In combination with microfluidics and other surface based technologies and spectroscopies, numerous basic cell biology and biotechnology applications are now possible with these dynamic substrates [46–64]. The cell migration memory results may open a new research area to decipher how the dynamic microenvironment influences cell motility in cancer cell biology and development.

## Materials and Methods

All the solvents for the synthetic procedures were HPLC grade. THF was distilled from sodium benzophenone under nitrogen before use. Absolute ethanol was purchased from Aaper Alcohol & Chemical Company. Flash chromatography was carried out using silica gel (230–400 mesh). All amino acids and resin were purchased from Anaspec, Inc. (La Jolla, CA). The chemical reagents were purchased from Sigma Aldrich and Acros and used as received.

### Synthesis of Alkanethiols

Alkanethiols terminated with hydroquinone groups and tetra(ethylene glycol) groups were prepared as previously described [19].

### Preparation of Monolayers

All gold substrates were prepared by electron-beam deposition of titanium (3 nm) and then gold (12 nm) on glass cover slips (7.5 cm × 2.5 cm). All gold coated glass substrates were cut into 1 cm^2^ pieces and washed with absolute ethanol. The substrates were immersed in an ethanolic solution containing the alkanethiols (1 mM) for 12 hours, and then cleaned with ethanol prior to each experiment.

### Electrochemical Measurements

All electrochemical experiments were performed using a Bioanalytical Systems CV–100W potentiostat. Electrochemistry on SAMs was performed in 1 M HClO_4_, using a platinum wire as the counter electrode, Ag/AgCl as reference, and the gold SAM substrate as the working electrode. All cyclic voltammograms were recorded at a scan rate of 50 mV/s.

### Solid-Phase Peptide Synthesis

All peptides were synthesized by automated solid phase peptide synthesis using the CS136XT Peptide Synthesizer (CS Bio Co., Menlo Park, CA).

#### Linear RGD

Fmoc (9-fluorenylmethoxycarbonyl)-protected amino acids were used on Fmoc-Ser(tBu)-Rink Amide-MBHA resin. Synthesized peptide was cleaved from the resin by agitating in a solution of trifluoroacetic acid (TFA):water:triisopropylsilane (95:2.5:2.5) for 3 hours. TFA was evaporated and the cleaved peptide was precipitated in cold diethyl ether. The water-soluble peptide was extracted with water and lyophilized. Mass spectral data confirmed the peptide product. MS (ESI) (m/z): [M+H]^+^ calculated for linear RGD-oxyamine (C_25_H_45_N_11_O_11_), 676.69; found, 676.5. [M+H]^+^ calculated for control scrambled peptide, GRD-oxyamine (C_25_H_45_N_11_O_11_), 676.69; found, 676.4. [M+H^+^] calculated for control soluble peptide RGD (C_17_H_31_N_9_O_8_), 490.48; found, 490.3.

#### Cyclic RGD

The peptide DfKRG was synthesized using H-Gly-OH preloaded 2-chlorotrityl resin. The mixture of acetic aicd:trifluoroethanol:dichloromethane (1:1:3) was added for cleavage. The resulted peptide was dissolved in DMF and added with N, N-diisopropylethylamine (DIEA) and PyBOP. The reaction was stirred for 12 hours and the solvent was removed in vacuum. The resulted cyclic peptide was treated with TFA:water:triisopropylsilane (95:2.5:2.5) for 3 hours and precipitated in diethyl ether. To introduce the oxyamine group on Lys side chain, the peptide was treated with BOC-aminooxy acetic acid, PyBOP and DIEA in DMF for 10 hours. After removing DMF, the peptide was added with TFA for 1 hour and precipitated in diethyl ether. The sample was dissolved in water and purified by HPLC (Waters). MS (ESI) (m/z): [M+H]^+^ calculated for cyclic RGD-oxyamine (C_29_H_44_N_10_O_9_), 677.72; found, 677.4.

### Cell Culture

The 3T3-Swiss albino cells (Tissue Culture Facility, UNC at Chapel Hill) were cultured in Dulbecco’s Modified Eagle’s Medium (Sigma) supplemented with 10% bovine calf serum (Hyclone) and 1% penicillin/streptomycin (100 units of penicillin/ 100 μg of streptomycin per mL, Gibco) at 37°C and 5% CO_2_. To detach cells from the culture flask, cells were rinsed with phosphate-buffered saline twice (PBS, sigma) and 0.05% trypsin /0.53 mM EDTA (Gibco) was added. After incubating for 5 minutes, cells were resuspended in serum-free medium and centrifuged at 1000 rpm for 5 minutes to remove trypsin. Cells were resuspended in serum-free medium and added onto the substrates for the experiments.

CHO cell lines B2a27, B2a10, and B2 were gift from Prof. Rudy Juliano. CHO cells were cultured in Minimum Essential Medium Containing GlutaMAX-I (L-Alanyl-L-Glutamine) substituted on a molar equivalent basis for L-glutamine (Gibco) supplemented with 10% fetal bovine serum (Hyclone) and 1% penicillin/streptomycin (Gibco) at 37°C and 5% CO_2_. The same procedure as above was used to detach cells from the culture flask.

### Preparation of Dynamic Surfaces

Microcontact printing was used to print hexadecanethiol on the gold-coated substrates in circular or linear patterns with a poly(dimethylsiloxane) stamp. The substrates were immersed in a 1 mM ethanol solution of hydroquinone-terminated alkanethiol and tetra(ethylene glycol)-terminated alkanethiol for 12 hours to make mixed SAMs. The substrates were rinsed with absolute ethanol and dried under air flow. The patterned regions with hexadecanethiolate monolayer were adsorbed with fibronectin (from bovine plasma, Sigma) by adding a solution of fibronectin in PBS (0.1 mg/mL) onto substrates. After 2 hours, the substrates were rinsed with water and dried. The 3T3-Swiss albino cells in serum-free medium were seeded onto the SAM substrates in a concentration of 10^5^ cells/mL and incubated at 37°C and 5% CO_2_. After 3 hours, the serum-free medium was replaced with serum-containing medium and incubated at the same condition. When cells were attached and relaxed evenly on the patterns (usually after 10–12 hours), the substrates were rinsed in serum-free medium twice. The substrates were oxidized electrochemically at 750 mV for 5 seconds. To the substrates was added 20 mM RGD-oxyamine peptide (linear, cyclic or mixture RGD) solution in serum-free medium and incubated at 37°C and 5% CO_2_ for 2 hours. The substrates were then placed in serum-containing medium and incubated at 37°C and 5% CO_2_ and monitored with live cell microscopy.

### Preparation of Non-dynamic Surfaces

Mixed SAMs were prepared by immersing gold-coated glass cover slips in a 1 mM ethanol solution of hydroquinone-terminated alkanethiol (1 to 10% in v/v) and tetra(ethylene glycol)-terminated alkanethiol for 12 hours. The substrates were rinsed with absolute ethanol and dried under air flow. The substrates were oxidized electrochemically at 750 mV for 10 seconds and rinsed with water. The substrates were incubated with 20 mM RGD (linear, cyclic or mixture RGD peptide) in PBS for 3 hours at room temperature. The substrates were then washed in water and dried. 3T3-Swiss albino fibroblasts were then added in the concentration of 10^4^ cells/mL. The low cell seeding density is in order to exclude interactions between cells.

### Staining for Fluorescence Microscopy

Adherent cells were fixed with 3.2% paraformaldehyde in PBS for 10 minutes and then permeabilized with 0.1% Triton-X100 in PBS (PBST) for 10 minutes. Cells were then stained with anti-paxillin anitibodies (1:200, BD biosciences) in PBST containing 5% goat serum for 1 hour, followed by Cy2-conjugated goat anti-mouse IgG (1:200 in PBST, Jackson ImmunoResearch), phalloidin-tetramethylrhodamine B isothiocyanate (1:100 in PBST, Sigma), and DAPI (4’, 6-diamidino-2-phenylindole dihydrochloride, Sigma) (1:500 in PBST) for 1 hour. Substrates were rinsed with deionized water before being mounted onto glass cover slips for microscopy. All optical and fluorescent micrographs were imaged using a Nikon inverted microscope (model TE2000–E). All images were captured and processed by MetaMorph.

### Movies

Time-lapse images were recorded by 2 or 5 minutes interval for 30–72 hours. The images were combined using MetaMorph software to create movie files. Cell tracking function in MetaMorph was used to measure the total distance cells had migrated on dynamic or non-dynamic surfaces.

### BrdU Staining

Actively growing cells on the dynamic surfaces were pulsed for 1 hour with 10 μM BrdU (bromodeoxyuridine, Sigma) at 37°C and 5% CO_2_. After washing with PBS, ice cold 70% ethanol was added and cells were incubated for 20 minutes at room temperature (RT). Cells were washed in PBS and then 2 M HCl was added and incubated at RT for 20 minutes. After rinsing with PBS, cells were treated with 0.1 M sodium borate (Na_2_B_4_O_7_, pH 8.5) for 2 minutes at RT. Then 0.5% BSA (bovine serum albumin) in PBST was added for 15 minutes. Anti-BrdU monoclonal antibody (BD pharmingen) in 0.5% BSA/PBST (1:500) was added for 1 hour at RT. Cells were washed in PBST and treated with Cy2-conjugated goat anti-mouse IgG (1:100) for 30 minutes at RT. Washing in PBST was followed. DAPI diluted in PBST (1:500) was added for 10 minutes to stain whole nuclei.

### Whole Genome Microarray

Dynamic and non-dynamic surfaces were prepared with linear RGD or cyclic RGD as described above. The peptide was immobilized in the density of 2% of the surface area. Cells were allowed to migrate for 24 hours in the incubator before collecting for RNA extraction. Cells on the fibronectin pattern were prepared with no addition of RGD ligand, but they were incubated for the equal amount of time with the non-dynamic substrates. As a reference, regularly cultured cells in tissue culture flask were used. Cells from 70~80% confluent monolayers with regular morphology and behavior were harvested to extract RNA. Total RNA from each sample and reference was purified using the RNeasy Mini Kit (Qiagen Inc., Valencia, CA, USA) according to the manufacturer's protocol. RNA quality check, labeling, array hybridization and image scanning were performed by the Genomics and Bioinformatics Core at the UNC Lineberger Comprehensive Cancer Center (Chapel Hill, NC, USA) and the following is a brief description of the method. The integrity of the RNA was determined using the 2100 Bioanalyzer RNA Series II Kits (Agilent Technologies Inc., Santa Clara, CA, USA). The spike mixes of Two-color RNA Spike-In kit (Agilent Technologies Inc.) were diluted, then added directly to RNA samples prior to amplification and labeling. Total RNA samples (2.0 μg) were amplified using T7 primers to make cRNA. The cRNAs from samples were labeled with Cy5 and the reference cRNA was incorporated with Cy3. The labeled samples and reference were co-hybridized to Agilent Whole Mouse Genome Microarrays (G4122F). They were then washed and scanned on an Agilent DNA microarray scanner (Agilent Technologies Inc.). Each microarray experiment was repeated three more times per sample. The microarray images generated by the scanner were analyzed using Feature Extraction 9.5.3 software (Agilent Technologies Inc.). The raw data tables were uploaded into the UNC Microarray Database where a Lowess normalization is automatically performed to adjust the Cy3 and Cy5 channels. All microarray raw data tables are available at Gene Expression Omnibus Accession number GSE62304 (http://www.ncbi.nlm.nih.gov/geo/). The Hierarchical cluster image was created by GeneSpring 7 (Agilent Technologies Inc.). Using this software the raw data was normalized by intensity dependent (Lowess) normalization (per spot and per chip). Among 41,233 genes, 1716 genes were selected by filtering the data with t-test p-value of four replicates less than 0.05 and normalized data larger than 2.5 in at least 1 of 5 sample conditions.

## Supporting Information

S1 Fig Cyclic voltammogram characterization of the electroactive monolayers. Mixed monolayer of alkanethiolates presenting hydroquinone (50%) and tetra(ethylene glycol) (50%) groups on gold was used as working electrode. Electrochemistry was performed at a scan rate of 50 mV/s in 1M HClO_4_. (A) The hydroquinone monolayer is reversibly oxidized to the quinone at 540 mV and reduced back at 320 mV. (B) The oxime conjugate with RGD peptide has characteristic peaks at 620 mV (oxidation) and 480 mV (reduction). The cyclic voltammograms were used to determine the extent and yield of the interfacial reaction.
(PDF)

S2 Fig Control experiment of dynamic substrates. (A) Non-patterned area was composed of tetra(ethylene glycol)- and hydroquinone- terminated alkanethiolates (99:1). No adhesive ligand was immobilized. Cells were found confined in the patterned area after 48 hrs. (B) GRD-oxyamine was immobilized in the same procedure as RGD surface. Cells were found confined in the patterned area after 48 hrs. Color: green, tubulin; red, actin; blue, nuclei.
(PDF)

S1 Movie Control experiment of dynamic substrates. Swiss albino 3T3 cells were patterned in circles (diameter 160 μm). Non-patterned area was composed of tetra(ethylene glycol)- and hydroquinone- terminated alkanethiolates (ratio of 99:1) and no adhesive ligand was immobilized. The frames were taken every 5 minute for 3 days. Cells near the edge of the pattern dynamically sample the environment by protruding filopodia and lamellipodia and membrane ruffling constantly. Cells do not migrate out and are remained confined because there is no ligand to interact with outside the patterned area.
(AVI)

S2 Movie Cell migration on linear RGD dynamic surface. Cells were patterned in the same manner as in Movie 1. The substrate was electrochemically activated and RGD peptide was immobilized on the surface in the presence of patterned cells. The time-lapse microscopy was started 4 hrs after RGD immobilization. The frames were taken every 5 minute for 3 days. Cells actively migrate off the pattern *via* interaction with RGD on the surface.
(AVI)

S3 Movie Cell migration on cyclic RGD dynamic surface. Cyclic form of RGD ligand was immobilized on the surface. The time-lapse images were collected in the same manner as in S2 Movie.
(AVI)

S1 Table Extended gene list from microarray analysis. (PDF)
