# Supplementary figures and images for: A Molecular Smart Surface for Spatio-Temporal Studies of Cell Mobility
Source: PLoS One. 2015 Jun 1;10(6):e0118126. doi: 10.1371/journal.pone.0118126 (PMC4452080; doi:10.1371/journal.pone.0118126)

A

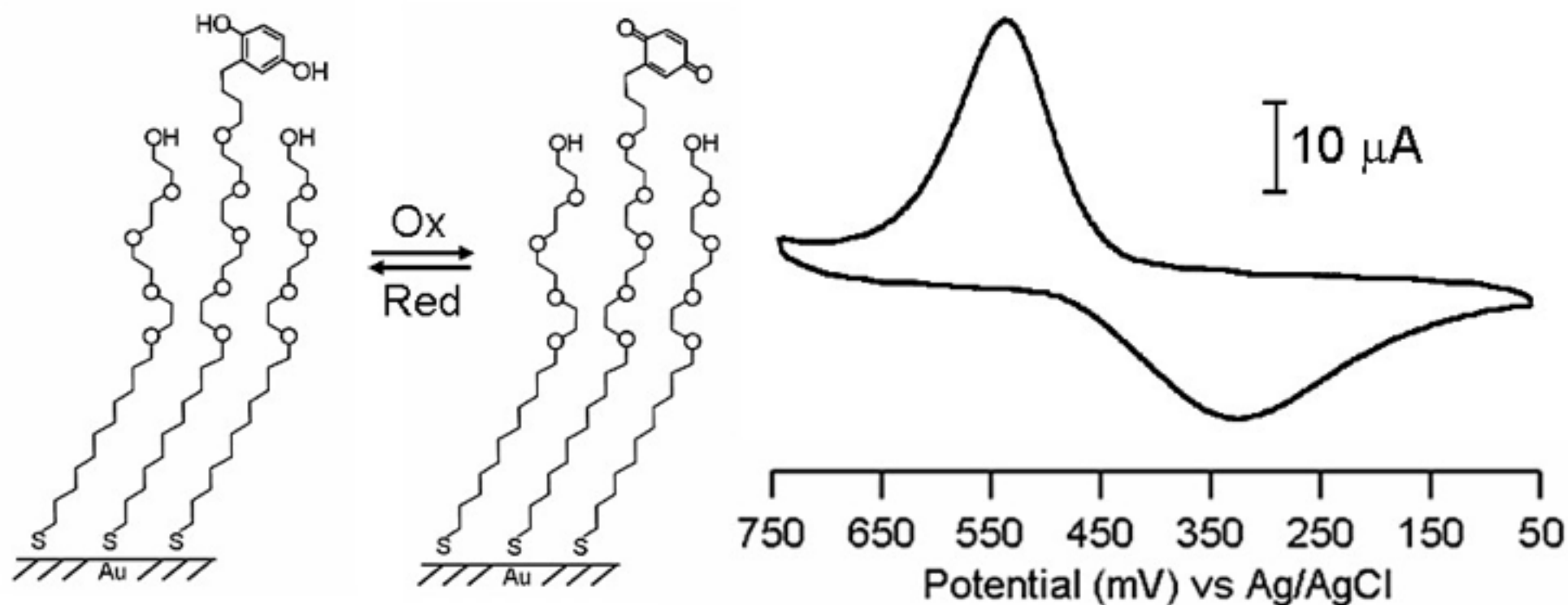

B

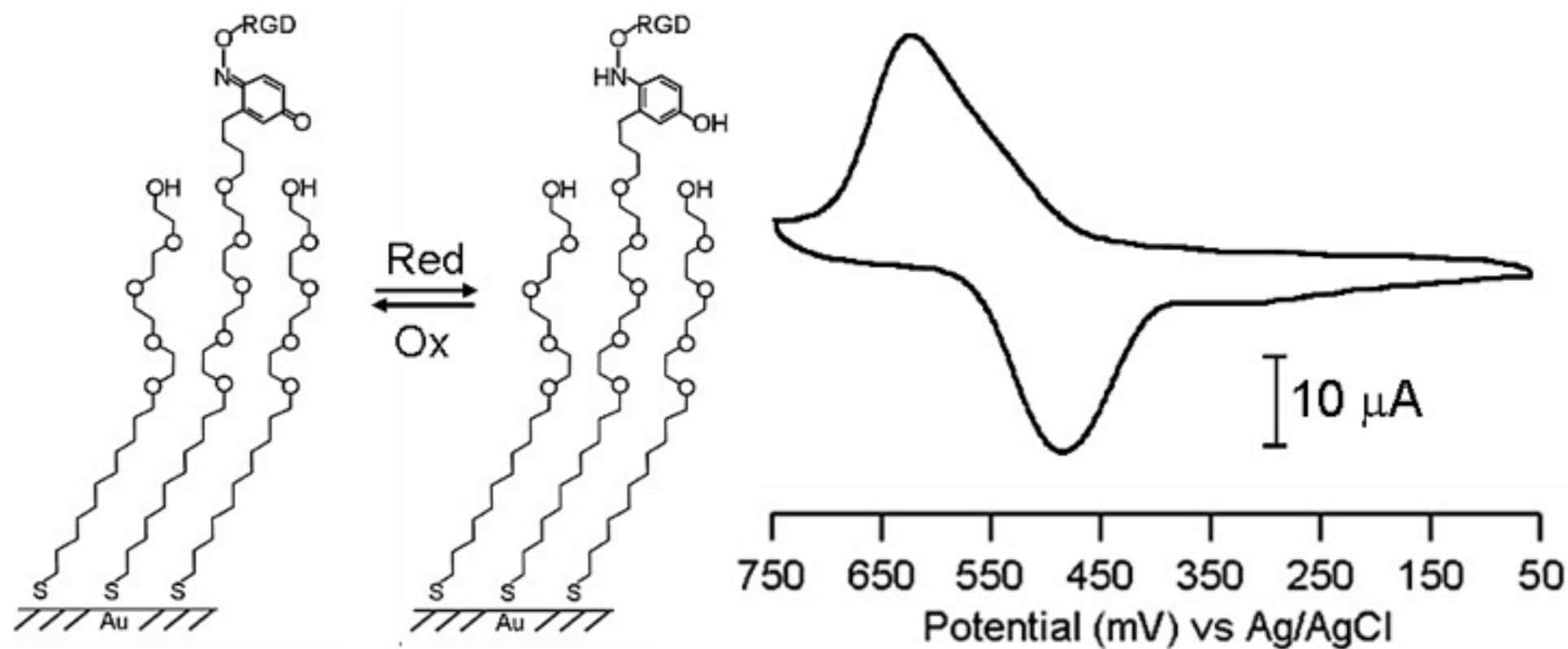

Supplement: S1 Fig — Mixed monolayer of alkanethiolates presenting hydroquinone (50%) and tetra(ethylene glycol) (50%) groups on gold was used as working electrode. Electrochemistry was performed at a scan rate of 50 mV/s in 1M HClO4. (A) The hydroquinone monolayer is reversibly oxidized to the quinone at 540 mV and reduced back at 320 mV. (B) The oxime conjugate with RGD peptide has characteristic peaks at 620 mV (oxidation) and 480 mV (reduction). The cyclic voltammograms were used to determine the extent and yield of the interfacial reaction. (PDF) [file pone.0118126.s001.pdf]

A

200  $\mu\text{m}$ 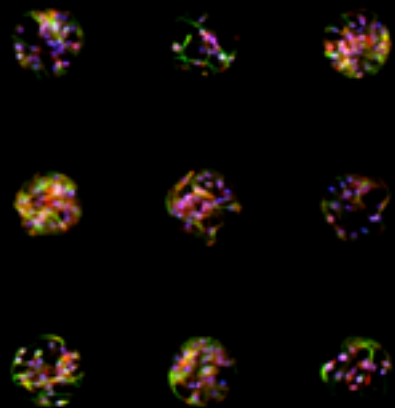

B

200  $\mu\text{m}$ 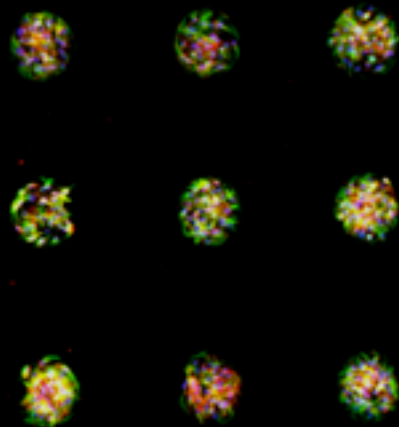

Supplement: S2 Fig — (A) Non-patterned area was composed of tetra(ethylene glycol)- and hydroquinone- terminated alkanethiolates (99:1). No adhesive ligand was immobilized. Cells were found confined in the patterned area after 48 hrs. (B) GRD-oxyamine was immobilized in the same procedure as RGD surface. Cells were found confined in the patterned area after 48 hrs. Color: green, tubulin; red, actin; blue, nuclei. (PDF) [file pone.0118126.s002.pdf]
