# Supplementary material for: A Molecular Smart Surface for Spatio-Temporal Studies of Cell Mobility
Source: PLoS One. 2015 Jun 1;10(6):e0118126. doi: 10.1371/journal.pone.0118126 (PMC4452080; doi:10.1371/journal.pone.0118126)
Supplement: S1 Table — (PDF) [file pone.0118126.s006.pdf]

Category	Dyn C	Dyn L	FN	ND C	ND L	Name	Symbol
Signaling	3.09	3.75	2.78	15.64	13.12	Wingless-related MMTV integration site 2	Wnt2
	4.32	1.11	2.86	6.03	1.24	Wingless-related MMTV integration site 3	Wnt3
	2.51	2.90	5.77	0.50	0.48	Wingless-related MMTV integration site 6	Wnt6
	1.43	1.31	1.23	3.32	3.67	Wingless-related MMTV integration site 11	Wnt11
	2.75	2.57	3.00	0.67	0.77	Secreted frizzled-related protein 1	Sfrp1
	2.80	3.08	7.29	0.31	0.21	Calcium channel, voltage-dependent, T type, alpha 1G subunit	Cacna1g
	2.37	2.37	4.91	0.60	0.69	RAS, guanyl releasing protein 2	Rasgrp2
	2.48	3.22	8.89	0.81	0.89	Axin2	Axin2
	3.35	3.81	1.46	23.96	15.28	Interleukin 1 alpha	Il1a
	2.70	2.97	3.10	1.20	1.11	Naked cuticle 2 homolog (Drosophila)	Nkd2
	1.99	2.27	5.78	0.18	0.19	Prostaglandin F receptor	Ptgfr
	3.21	3.54	3.81	7.96	9.22	Sphingosine kinase 1	Sphk1
	1.73	1.94	3.00	1.84	1.75	Rho-associated coiled-coil containing protein kinase 1	Rock1
	0.97	0.91	0.93	1.36	1.10	Ras homolog gene family, member A	Rhoa
	2.42	3.52	5.68	1.37	1.11	Patched homolog 2	Ptch2
	3.56	3.13	7.75	0.18	0.27	Gastrin releasing peptide receptor	Grpr
	1.31	1.81	1.17	15.94	14.69	Amphiregulin	Areg
Focal Adhesion/ Regulation of actin	4.05	3.09	5.99	9.48	15.43	Von Willebrand factor homolog	Vwf
	3.09	3.09	3.62	13.86	10.71	NCK associated protein 1 like	Nckap1l
	1.54	0.75	2.55	0.28	0.23	Insulin-like growth factor 1	Igf1
	1.65	1.88	0.50	0.46	0.29	Actin, alpha 2, smooth muscle, aorta	Acta2
	0.83	1.87	1.08	0.93	1.76	Myosin, heavy polypeptide 9, non-muscle	Myh9
	0.67	0.32	0.42	0.49	0.55	Vav 3 oncogene	Vav3
	2.03	2.50	0.90	5.60	4.09	Myosin, light polypeptide 7, regulatory	Myl7
Integrins	7.32	8.38	13.85	1.12	0.63	Integrin, alpha 11	Itga11
	1.50	1.92	1.64	5.39	7.78	Integrin alpha 2	Itga2
	0.97	0.85	0.43	0.23	0.28	Integrin alpha 1	Itga1
	2.27	1.97	2.44	1.01	0.82	Integrin alpha 9	Itga9
	1.04	0.98	0.56	1.80	2.85	Integrin alpha 5 (fibronectin receptor alpha)	Itga5
	1.24	1.02	0.62	1.25	1.57	Integrin beta 1 (fibronectin receptor beta)	Itgb1
	1.32	1.44	1.78	1.08	1.45	Integrin beta 5	Itgb5
	1.84	1.16	1.12	1.96	1.75	Integrin beta 3	Itgb3
Adhesion Molecules	1.34	1.54	3.02	0.64	0.48	Intercellular adhesion molecule	Icam1
	0.80	1.07	2.07	3.38	3.30	Neogenin	Neo1
	0.88	0.86	2.42	9.45	9.69	Cadherin EGF LAG seven-pass G-type receptor 1	Celsr1
	1.50	1.80	1.30	5.38	5.56	Cadherin 5	Cdh5
	2.71	2.81	3.57	10.13	8.98	Junction adhesion molecule 2	Jam2
